# Clinically recommended strategies for nutritional metabolic intervention in cardiac surgery: a narrative review of oral support agents and modulators on postoperative outcomes and mechanisms

**DOI:** 10.3389/fnut.2026.1929844

**Published:** 2026-08-24

**Authors:** Bo Kong, Yan Yang, Min Song, Meng Yan, Yanhai Meng

**Affiliations:** Fuwai Hospital, Chinese Academy of Medical Sciences and Peking Union Medical College, Beijing, China

**Keywords:** cardiac surgery, evidence-based practice, myocardial protection, nutritional supplements, perioperative period

## Abstract

**Background:**

The burden of postoperative complications following cardiac surgery remains substantial, and nutritional metabolic intervention represents an important adjunctive strategy.

**Aim:**

To systematically evaluate the evidence base for oral nutritional supplements and their clinical application strategies.

**Methods:**

A comprehensive literature search was conducted in PubMed, Embase, Cochrane Library, and Web of Science from inception to June 2026. Randomized controlled trials (RCTs), meta-analyses, and systematic reviews on the perioperative use of nutritional supplements in cardiac surgery were included.

**Results:**

A total of 89 relevant studies were identified and synthesized narratively. Based on the quality of available evidence and clinical benefit, the supplements were categorized as follows: Strongly recommended: magnesium; Recommended: L-carnitine; Moderate/promising support: coenzyme Q10, vitamin C, melatonin, D-ribose, citrulline, glutamine; Not recommended/insufficient evidence: creatine/phosphocreatine, taurine, thiamine, vitamin D (unless baseline deficiency), dietary nitrates, N-acetylcysteine; Emerging/experimental (pending evaluation): NAD + precursors, PQQ, astaxanthin, quercetin, curcumin, carnosine, ergothioneine; Use with caution/contraindicated: ginseng, red yeast rice, probiotics (in specific populations).

**Conclusion:**

Magnesium should be considered a routine adjunctive therapy, while L-carnitine and coenzyme Q10 are recommended supplements. Individualized selection is critical, requiring consideration of patient characteristics, surgical type, and baseline status. However, it should be noted that the current evidence base does not yet support biomarker-driven or genotype-guided precision supplementation; future high-quality RCTs are needed to validate the efficacy of these supplements across different patient subsets. The translational gap from preclinical studies to clinical application should be carefully addressed, and more high-quality RCTs are needed to validate the efficacy of emerging supplements.

## 1 Introduction

The rapid advancement of cardiac surgery and the widespread application of cardiopulmonary bypass (CPB) technology have made increasingly complex cardiac procedures feasible. However, the CPB process inevitably triggers systemic inflammatory responses and ischemia–reperfusion injury (IRI), leading to a persistently high incidence of postoperative complications (1). Common complications include postoperative atrial fibrillation (POAF, incidence 20–50%), low cardiac output syndrome, acute kidney injury (AKI), stroke, and cognitive dysfunction. Among these, CPB duration has been identified as an independent risk factor for central nervous system complications (2). These complications not only prolong hospital stays and increase healthcare costs but also significantly affect both short- and long-term patient outcomes (3, 4). Current pharmacological strategies (e.g., *β*-blockers, statins, ACEIs/ARBs) have demonstrated certain efficacy in preventing POAF and providing myocardial protection; nevertheless, a subset of patients remain nonresponsive or intolerant to these treatments. Consequently, the search for safe, effective, and cost-effective adjunctive intervention strategies has become a research hotspot in cardiac surgery. Nutritional metabolic intervention, as an adjunctive approach, offers advantages such as multi-target effects, low side-effect profile, and low cost, and has thus gained increasing attention in recent years (1).

Myocardial IRI sustained during CPB represents the core pathophysiological mechanism underlying postoperative complications (5). The development of IRI involves three interrelated mechanisms: (1) Energy metabolic failure: ATP depletion in cardiomyocytes during the ischemic phase, coupled with delayed mitochondrial functional recovery after reperfusion, leads to reduced myocardial contractility and an increased risk of arrhythmias (6). (2) Oxidative stress (ROS burst): Reperfusion induces massive production of reactive oxygen species (ROS) that overwhelms the capacity of endogenous antioxidant systems, resulting in lipid peroxidation, protein denaturation, DNA damage, and further mitochondrial dysfunction (1). (3) Inflammatory response: Ischemia–reperfusion activates inflammatory pathways such as NF-κB, leading to the substantial release of pro-inflammatory cytokines (TNF-*α*, IL-1β, IL-6, etc.), which trigger an inflammatory cascade and amplify tissue injury (7). These three mechanisms are closely intertwined, with mitochondria serving as both the primary source of ROS and the center of energy metabolism. Therefore, mitochondrial-targeted nutritional metabolic intervention represents a key direction for precision therapy (8). Recent studies have shown that CPB leads to reduced myocardial NAD + levels and impaired PGC-1α signaling, and that supplementation with NAD + precursors (e.g., nicotinamide) effectively reverses mitochondrial dysfunction, providing a theoretical basis for mitochondrial-targeted interventions (9).

The concept of individualized intervention in nutritional supplementation implies that not all patients require all supplements; the efficacy of the same supplement may vary across populations, and benefits must be weighed against risks (including drug interactions, adverse effects, and costs) (10, 11).

Existing RCTs and meta-analyses exhibit considerable heterogeneity, with substantial variability in the efficacy of the same supplement across different studies. For example, the organ-protective effects of nitric oxide have been inconsistent across studies, showing efficacy for AKI but not for other endpoints, suggesting that intervention timing and dosage may be key modifying factors (12). This heterogeneity may stem from differences in patient baseline characteristics (age, nutritional status, comorbidities), surgical type and complexity, supplement formulation, dosage, timing and duration of administration, selection of outcome measures and follow-up duration, as well as small sample sizes and insufficient statistical power. Therefore, constructing an evidence-based reference framework based on available evidence to guide individualized clinical application is critical for improving efficacy, reducing adverse effects, and optimizing healthcare resource utilization.

This review aims to systematically evaluate the evidence base for commonly used oral nutritional supplements in the perioperative period of cardiac surgery, and to provide an evidence-based reference framework based on evidence quality, clinical benefit, safety, and cost-effectiveness, with the goal of informing clinical practice.

## 2 Literature search strategy

### 2.1 Search strategy

A comprehensive literature search was conducted in PubMed, Embase, Cochrane Library, and Web of Science from inception to June 2026, using a combination of MeSH terms and free-text words. Taking PubMed as an example, the full search strategy was: (“cardiac surgery” OR “heart surgery” OR “cardiovascular surgery” OR “cardiopulmonary bypass” OR “CPB”) AND (“nutritional supplements” OR “dietary supplements” OR “perioperative” OR “peri-operative” OR “postoperative complications”) AND (“magnesium” OR “coenzyme Q10” OR “ubiquinone” OR “L-carnitine” OR “carnitine” OR “N-acetylcysteine” OR “acetylcysteine” OR “vitamin C” OR “ascorbic acid” OR “melatonin” OR “glutamine” OR “D-ribose” OR “citrulline” OR “creatine” OR “phosphocreatine” OR “taurine” OR “vitamin D” OR “thiamine”). Equivalent search strategies adapted to the syntax of Embase, Cochrane Library, and Web of Science were also applied.

Due to the narrative nature of this review, we did not perform a formal systematic review with duplicate independent screening, PRISMA flow diagram, or quantitative risk-of-bias assessment. Instead, we synthesized evidence narratively, with evidence quality and recommendation strength presented in Table 1.

**Table 1:** Recommendation grades for perioperative nutritional supplements in cardiac surgery.

Recommendation grade	Evidence quality	Clinical benefit	Safety	Representative supplements
Strongly recommended	High	Clear	Good	Magnesium
Recommended	Moderate	Clear	Good	L-carnitine
Moderate/promising support	Moderate or low	Potential benefit	Good	coenzyme Q10, Vitamin C, melatonin, D-ribose, citrulline, glutamine
Not recommended/insufficient evidence	Low or inconsistent	Unclear	Good	NAC, Creatine/phosphocreatine, taurine, thiamine, vitamin D (unless baseline deficiency), dietary nitrates
Emerging/experimental	Very low (animal/small-scale studies only)	To be verified	To be evaluated	NAD + precursors, PQQ, astaxanthin, quercetin, curcumin, carnosine, ergothioneine
Contraindicated/use with caution	-	-	Safety concerns	Ginseng (bleeding risk), red yeast rice (drug interaction), probiotics (infection risk in immunocompromised patients)

### 2.2 Inclusion and exclusion criteria

Inclusion criteria: (1) Study types: randomized controlled trials (RCTs), systematic reviews, and meta-analyses; (2) Study population: adult patients (≥18 years of age) undergoing cardiac surgery (including CABG, valve surgery, and great vessel surgery); (3) Intervention: perioperative use of oral or intravenous nutritional supplements; (4) Outcome measures: primary outcomes included postoperative atrial fibrillation, myocardial infarction, mortality, and length of hospital stay; secondary outcomes included biochemical markers (troponin, CK-MB, BNP, etc.), cardiac function indicators (EF, CI, etc.), and inflammatory response markers (CRP, IL-6, TNF-*α*, etc.). Exclusion criteria: (1) Non-cardiac surgery; (2) Case reports, narrative reviews, conference abstracts, or animal studies; (3) Incomplete data or data that could not be extracted. Both primary RCTs and high-quality systematic reviews/meta-analyses were included. For supplements with available meta-analyses, we prioritized their conclusions; for those without, we referenced primary RCTs directly. Supplements were selected for inclusion if they are commonly used in perioperative clinical practice and have direct published evidence (RCTs, meta-analyses, or systematic reviews) specific to the adult cardiac surgery population. Agents with evidence exclusively from non-cardiac surgical populations or from the general cardiovascular prevention setting were not included unless sufficient cardiac surgery-specific data were available.

### 2.3 Study selection process

The initial search yielded 130 records across the four databases. Following deduplication and title/abstract screening, full-text assessment was performed on 102 records, yielding 89 studies (including RCTs, meta-analyses, and systematic reviews) that were deemed relevant for narrative synthesis.

## 3 Main body: evidence-based evaluation of various supplements

A wide variety of nutritional supplements are used during the perioperative period of cardiac surgery, with diverse mechanisms of action. In general, they can be categorized into the following major classes: energy metabolism modulators (e.g., coenzyme Q10, D-ribose, L-carnitine, creatine/phosphocreatine), electrolytes (e.g., magnesium, taurine), antioxidants and anti-inflammatory agents (e.g., vitamin C, N-acetylcysteine, melatonin), trace minerals (e.g., selenium, zinc), immunonutrition and amino acids (e.g., glutamine, immunonutrition, citrulline), NO pathway modulators (e.g., adenosine, nitrates/beetroot juice), fat-soluble vitamins (e.g., vitamin D, thiamine), and emerging supplements (e.g., NAD + precursors, PQQ, astaxanthin, quercetin, curcumin, carnosine, ergothioneine). These supplements exert myocardial protective effects through distinct molecular mechanisms, including improving energy metabolism, reducing oxidative stress, inhibiting inflammatory responses, modulating mitochondrial function, and enhancing vascular endothelial function. However, the quality of available evidence-based data varies considerably, ranging from high-quality meta-analyses to small-scale proof-of-concept studies. Therefore, this review systematically evaluates each category of supplement based on the quality of available evidence, degree of clinical benefit, safety, and cost-effectiveness, and proposes recommendations for clinical application. The following sections are organized according to this classification framework.

**Figure 1:**
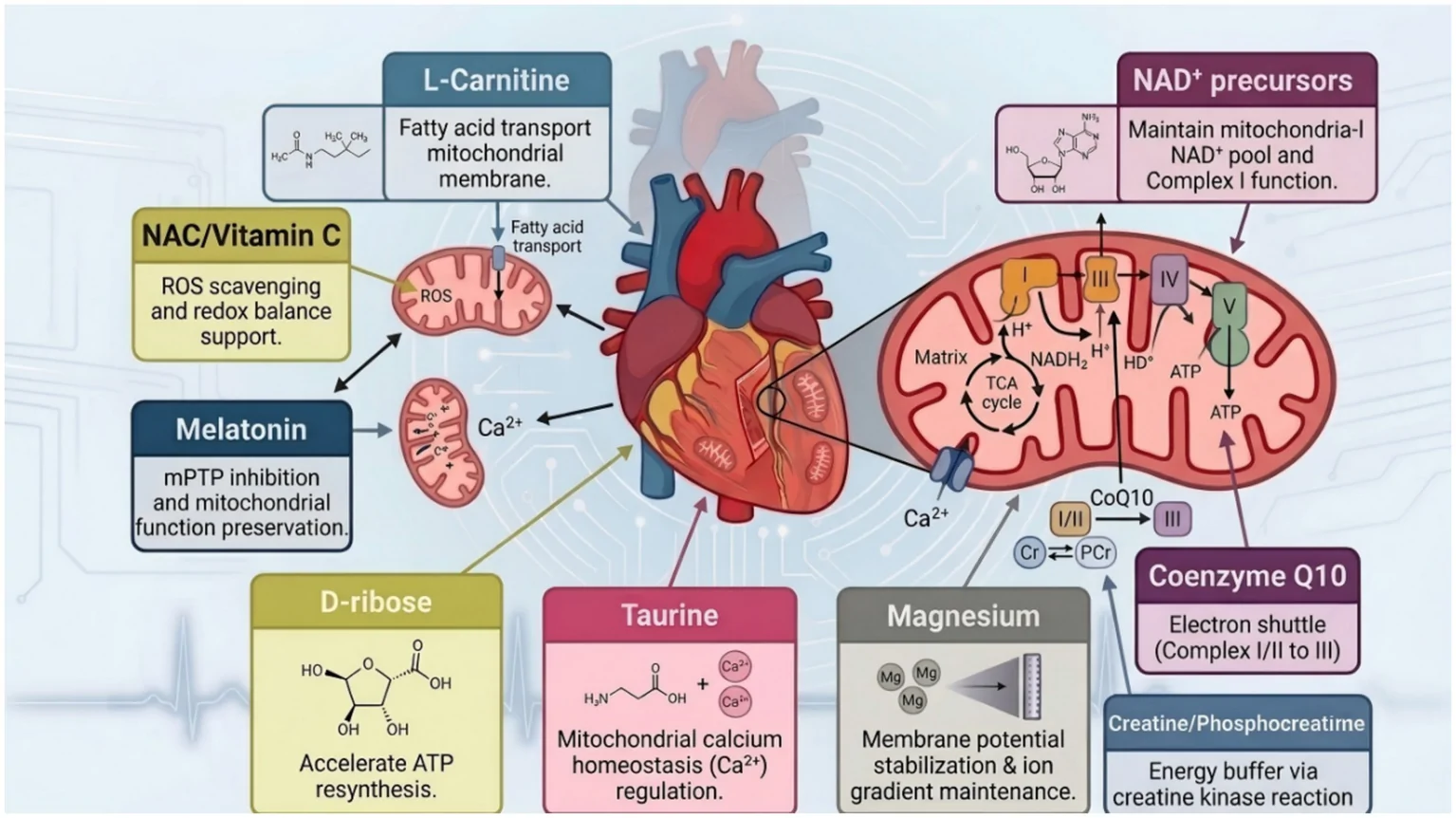
Sites of action of perioperative nutritional supplements targeting mitochondrial energy metabolism during cardiac surgery.

The major sites of action of these supplements within the mitochondrial metabolic network are illustrated in Figure 1. Among the energy metabolism modulators, L-carnitine facilitates the transport of long-chain fatty acids into the mitochondrial matrix for *β*-oxidation, coenzyme Q10 serves as an electron shuttle between Complex I/II and Complex III in the electron transport chain (ETC), D-ribose accelerates ATP resynthesis, and creatine/phosphocreatine acts as an energy buffer via the creatine kinase reaction. Meanwhile, magnesium stabilizes the mitochondrial membrane potential, taurine regulates intracellular calcium homeostasis, and melatonin, L-carnitine, and creatine inhibit the opening of the mitochondrial permeability transition pore (mPTP). Additionally, NAC (as a glutathione precursor) and vitamin C scavenge reactive oxygen species (ROS) within the matrix, while NAD + precursors replenish NAD + to sustain ETC function. This integrated view highlights the complementary mechanisms of these supplements and supports the rationale for combination strategies that target multiple nodes of the mitochondrial pathway to optimize myocardial protection during cardiac surgery.

### 3.1 Electrolytes

#### 3.1.1 Magnesium

Magnesium is an essential cation in the human body, participating in more than 300 enzymatic reactions and playing a critical role in maintaining the electrophysiological stability and metabolic function of cardiomyocytes. Its cardioprotective mechanisms mainly include: (1) regulating the action potential duration of cardiomyocytes and stabilizing the membrane potential, thereby inhibiting the formation of ectopic pacemaker sites; (2) acting as an endogenous calcium antagonist that competitively inhibits calcium channels and alleviates ischemia–reperfusion-induced calcium overload; (3) improving vascular endothelial function and promoting nitric oxide-mediated vasodilation; (4) inhibiting nuclear factor-κB (NF-κB) activation, reducing the release of pro-inflammatory cytokines, and lowering oxidative stress levels (13–15).

Among the currently available perioperative nutritional supplements in cardiac surgery, magnesium has the most robust evidence base and the highest recommendation level. Multiple meta-analyses have consistently demonstrated that perioperative magnesium supplementation significantly reduces the incidence of postoperative atrial fibrillation (POAF). A meta-analysis published in 2023, which included 22 randomized controlled trials with a total of 2,147 patients, showed that the incidence of POAF was significantly lower in the magnesium group compared with the control group (OR = 0.32, 95%CI 0.24–0.43, *p* < 0.001), and the risk of postoperative ventricular arrhythmias was also significantly reduced (OR = 0.42, 95%CI 0.28–0.63, *p* < 0.001) (16). A specific analysis focusing on patients undergoing coronary artery bypass grafting (CABG) further confirmed that magnesium supplementation reduces the risk of POAF by 23–36% (17, 18).

Based on the above evidence, magnesium is recommended for all patients undergoing cardiac surgery, particularly those at risk of hypomagnesemia, including patients with diabetes mellitus, those on long-term diuretic therapy, and elderly patients. In clinical practice, hypomagnesemia should be corrected preoperatively. A commonly used regimen includes oral magnesium 500–1,000 mg on the evening before surgery, followed by continued intravenous supplementation during and after surgery. Magnesium has a favorable safety profile; common adverse effects include diarrhea with oral formulations and transient hypotension and nausea with rapid intravenous infusion. Severe hypermagnesemia is rare but should be avoided in patients with renal insufficiency, in whom magnesium accumulation may occur. Based on the available evidence, magnesium should be considered a routine adjunctive therapy in the perioperative period of cardiac surgery, with a strong recommendation.

### 3.2 Energy metabolism modulators

This class of supplements directly targets myocardial energy homeostasis, which is severely disrupted during ischemia–reperfusion. They act by enhancing ATP synthesis, facilitating substrate utilization, or buffering energy fluctuations, thereby improving myocardial contractile recovery and reducing stunning.

#### 3.2.1 L-carnitine

L-carnitine is an essential cofactor for the *β*-oxidation of long-chain fatty acids, functioning to transport long-chain fatty acids into the mitochondrial matrix for oxidative energy production (19). During cardiac ischemia–reperfusion, L-carnitine exerts myocardial protective effects through the following mechanisms: (1) improving myocardial energy metabolism and promoting ATP production; (2) reducing the accumulation of cardiotoxic intermediate products of fatty acid metabolism (e.g., long-chain acylcarnitines); (3) stabilizing the cardiomyocyte membrane and inhibiting the formation of ectopic pacemaker sites, thereby reducing the occurrence of arrhythmias; and (4) inhibiting the opening of the mitochondrial permeability transition pore (mPTP) and attenuating mitochondria-mediated apoptosis (20).

A meta-analysis of 13 RCTs comprising 786 patients (21) demonstrated that perioperative L-carnitine supplementation significantly improved postoperative hemodynamic parameters following cardiac surgery. Specifically, the L-carnitine group showed a significant increase in cardiac index (CI) compared with the control group (WMD = 0.45 L/min/m^2^, 95%CI 0.28–0.62, *p* < 0.001), a significant improvement in left ventricular ejection fraction (LVEF; WMD = 3.2, 95%CI 1.8–4.6%, *p* < 0.001), and a significant increase in left ventricular stroke work index (LVSWI). Furthermore, the incidence of postoperative atrial fibrillation was significantly reduced in the L-carnitine group (RR = 0.53, 95%CI 0.30–0.94, *p* = 0.03). The effect on all-cause mortality did not reach statistical significance but showed a trend toward reduction.

Based on available evidence, L-carnitine is recommended for the following patient populations: (1) those with preoperative myocardial energy metabolism disorders (e.g., dilated cardiomyopathy, chronic heart failure); (2) those undergoing complex cardiac surgery with an anticipated CPB duration > 2 h; and (3) those experiencing intraoperative hemodynamic instability requiring inotropic support. The recommended regimen is oral L-carnitine 1–2 g/day for 3–5 days preoperatively, addition to cardioplegic solution (2–4 g/L) intraoperatively, and continued intravenous supplementation of 1–2 g/day for 3–5 days postoperatively. L-carnitine has a favorable safety profile, with rare adverse effects including nausea, abdominal pain, and body odor change (fishy odor). It is contraindicated in patients with known hypersensitivity to L-carnitine. Based on the available evidence, L-carnitine is supported by moderate-quality evidence with clear clinical benefit, and the strength of recommendation is “recommended.”

#### 3.2.2 Coenzyme Q10 (CoQ10)

Coenzyme Q10 is an essential electron carrier between complexes I and III of the mitochondrial electron transport chain and also possesses potent antioxidant properties (22). In the perioperative period of cardiac surgery, the cardioprotective mechanisms of CoQ10 mainly include: (1) improving mitochondrial electron transfer efficiency and promoting ATP synthesis; (2) directly scavenging reactive oxygen species (ROS) and attenuating oxidative stress injury; (3) stabilizing the cardiomyocyte membrane structure and maintaining intracellular calcium homeostasis; and (4) inhibiting the release of inflammatory factors and attenuating ischemia–reperfusion-induced inflammatory responses.

Current clinical evidence is primarily derived from small-sample randomized controlled trials. An RCT by Rosenfeldt et al. (23) involving 121 patients showed that preoperative oral CoQ10 (300 mg/day for 2 weeks) significantly improved myocardial mitochondrial function (*p* < 0.01) but did not significantly affect postoperative hemodynamic parameters. A study by Makhija et al. (24) found that CoQ10 reduced the occurrence of reperfusion arrhythmias and shortened hospital stay. Studies by Chello et al. (25) and Taggart et al. (26) suggested that a short-term, high-dose CoQ10 loading regimen administered 1–2 days preoperatively provided some protective effects against reperfusion injury. Overall, large-scale, multicenter RCT evidence supporting the routine clinical application of CoQ10 is currently lacking.

Based on available evidence, CoQ10 is recommended for the following patient populations: (1) those with preexisting heart failure or cardiomyopathy; (2) those on long-term statin therapy (statins inhibit endogenous CoQ10 synthesis); and (3) elderly patients (endogenous CoQ10 levels decline with age). The recommended regimen is to begin supplementation at least 7–14 days preoperatively at a dose of 150–300 mg/day, preferably using a lipid-soluble formulation to enhance bioavailability, and to continue supplementation for 4–8 weeks postoperatively. CoQ10 has a favorable safety profile, with rare adverse effects including nausea, diarrhea, and decreased appetite. It should be noted that CoQ10 may potentiate the anticoagulant effect of warfarin; thus, close monitoring of the international normalized ratio (INR) is recommended when used concomitantly. Based on the available evidence, CoQ10 is supported by moderate-quality evidence from small-scale RCTs with mechanistically plausible benefit, but lacks confirmation from large multicenter trials. Therefore, the strength of recommendation is ‘moderate/promising support,’ provided that adequate preoperative preparation time (at least 1–2 weeks) is ensured and patients are appropriately selected (e.g., those on long-term statin therapy or with preexisting heart failure).

#### 3.2.3 D-ribose

D-Ribose is a rate-limiting precursor molecule for ATP synthesis. Following myocardial ischemia–reperfusion, insufficient endogenous D-ribose synthesis leads to delayed ATP recovery (27). Exogenous D-ribose supplementation promotes myocardial recovery through the following mechanisms: (1) accelerating the resynthesis of ATP and ADP; (2) improving myocardial energy metabolism; (3) promoting functional recovery of stunned myocardium; and (4) reducing cardiomyocyte apoptosis.

Current clinical evidence is primarily derived from small-sample studies and one relatively large-scale randomized controlled trial. Wyatt et al. (28) found in a cardioplegia solution experiment that the addition of D-ribose significantly improved postoperative cardiac function. Vance et al. (29) reported that perioperative parenteral D-ribose supplementation in patients undergoing aortic valve replacement maintained ejection fraction (EF). Perkowski et al. (30) conducted an RCT including 366 patients undergoing off-pump coronary artery bypass grafting (off-pump CABG), showing that oral D-ribose (5 g per dose, once daily on the day before surgery and for 3 days postoperatively, three times daily) significantly improved cardiac index (CI improvement 37% vs. 17% in the control group, *p* = 0.02) and reduced the incidence of postoperative complications and mortality. Although this study had a relatively large sample size, multicenter, large-sample RCTs are still lacking to further validate the routine application value of D-ribose in the perioperative period of cardiac surgery.

Based on available evidence, D-ribose is recommended for the following patient populations: (1) those with poor preoperative cardiac function (left ventricular ejection fraction < 40%); (2) those undergoing complex CABG surgery with a high anticipated risk of myocardial stunning; and (3) those exhibiting signs of inadequate myocardial protection during surgery (e.g., persistent ST-segment elevation on electrocardiogram, abnormal myocardial color). The recommended regimen is oral D-ribose (5 g three times daily) starting 1–3 days preoperatively and continuing for 3–5 days postoperatively; intravenous formulations may be considered for use under intensive care unit (ICU) monitoring. D-ribose has a favorable safety profile, with the main adverse effects being gastrointestinal discomfort (nausea, bloating). It should be noted that D-ribose should be used with caution or avoided in the early phase of acute myocardial ischemia, as it may exacerbate the “coronary steal” phenomenon in ischemic myocardium. Based on the available evidence, D-ribose is supported by moderate-quality evidence with potential benefit in specific populations, and the strength of recommendation is “consider use.”

#### 3.2.4 Creatine/phosphocreatine

Creatine/phosphocreatine is an essential component of the myocardial energy buffering system, rapidly regenerating ATP through the creatine kinase (CK) reaction and maintaining intracellular energy homeostasis (31). In the perioperative period of cardiac surgery, its potential myocardial protective mechanisms mainly include: (1) improving myocardial energy metabolism and buffering fluctuations in ATP levels; (2) stabilizing the integrity of the cardiomyocyte membrane; (3) attenuating myocardial stunning after reperfusion; and (4) inhibiting the opening of the mitochondrial permeability transition pore (mPTP).

Early small-sample randomized controlled trials suggested that phosphocreatine improves myocardial energy metabolism and reduces troponin release. However, a recent large-scale RCT (32) demonstrated that a multi-dose phosphocreatine regimen administered preoperatively, intraoperatively, and postoperatively did not significantly improve troponin release, renal function, or ICU/hospital length of stay. The discrepancy between this study and previous positive findings may be attributed to differences in patient baseline characteristics, CPB management strategies, and dosing regimens, suggesting that the current evidence is insufficient to support routine perioperative application of phosphocreatine.

Based on available evidence, phosphocreatine is not recommended as a routine perioperative supplement. Its use may only be considered in the following specific circumstances: (1) patients with preoperative severe heart failure in whom phosphocreatine is already part of the inotropic support regimen; (2) patients undergoing complex cardiac surgery with anticipated slow postoperative cardiac function recovery. Phosphocreatine has a favorable safety profile, with rare adverse effects including occasional reports of hypotension and arrhythmias. Based on the available evidence, the most recent high-quality RCT yielded negative results, and therefore routine use is not recommended.

#### 3.2.5 Taurine

Taurine is a sulfur-containing amino acid with pleiotropic cardioprotective effects (33). Its mechanisms mainly include: (1) regulating intracellular calcium homeostasis; (2) inhibiting oxidative stress and inflammatory responses through the Akt/caspase-9 signaling pathway, thereby reducing apoptosis; (3) stabilizing the cardiomyocyte membrane and cytoskeletal structure; and (4) modulating ion channel function.

Although the cardioprotective mechanisms of taurine are well established in basic research, human perioperative intervention trials are almost nonexistent. Early studies by Lombardini demonstrated elevated plasma taurine levels after cardiovascular surgery, but clinical trial data on exogenous perioperative taurine supplementation are extremely limited (34). Mechanistic studies by Suleiman et al. (35) described myocardial amino acid changes during ischemia–reperfusion but lacked interventional RCTs. Ex vivo experiments by Takatani et al. (36) confirmed the protective effect of taurine against H₂O₂-induced cardiomyocyte apoptosis. Current relevant evidence is primarily derived from animal experiments and small observational studies, and high-quality randomized controlled trials (RCTs) supporting its clinical application in the perioperative period of cardiac surgery are lacking. Therefore, the available evidence does not allow for a clear clinical recommendation.

Based on available evidence, there is currently insufficient justification for routine use of taurine in the perioperative period of cardiac surgery. Well-designed, adequately powered RCTs are needed in the future to evaluate its clinical efficacy and safety. Based on the available evidence, taurine has insufficient evidence and is not recommended for routine use.

### 3.3 Antioxidants and anti-inflammatory agents

Oxidative stress and inflammatory cascades are central to ischemia–reperfusion injury. Supplements in this category directly scavenge reactive oxygen species, replenish endogenous antioxidants, or inhibit pro-inflammatory signaling pathways, thereby mitigating tissue damage.

#### 3.3.1 N-acetylcysteine (NAC)

NAC is a precursor of glutathione (GSH) and possesses potent antioxidant and anti-inflammatory properties (37). In the perioperative period of cardiac surgery, its potential myocardial protective mechanisms mainly include: (1) scavenging reactive oxygen species (ROS) and attenuating oxidative stress injury; (2) inhibiting the NF-κB signaling pathway and reducing the release of pro-inflammatory cytokines; (3) improving vascular endothelial function and promoting vasodilation; and (4) inhibiting platelet aggregation.

Current clinical evidence is inconsistent regarding the overall organ-protective effects of NAC. A large meta-analysis of 29 RCTs comprising 2,486 patients (38) showed that perioperative NAC did not significantly improve major outcomes such as mortality, renal function, or cardiac function. However, a specific analysis focusing on the prevention of postoperative atrial fibrillation after CABG (39) found that NAC significantly reduced the incidence of postoperative atrial fibrillation (OR = 0.57, 95%CI 0.34–0.95, *p* = 0.04) and shortened hospital length of stay (MD = −0.66 days, *p* = 0.02). Further analysis suggested that the efficacy of NAC may be dose- and timing-dependent, with better effects observed with high doses (>100 mg/kg) and preoperative initiation.

Based on available evidence, NAC is recommended for CABG patients, particularly those at high risk for postoperative atrial fibrillation (e.g., elderly patients, those with a history of atrial fibrillation, or left atrial enlargement). The recommended regimen is to initiate NAC preoperatively (600–1,200 mg/day, oral or intravenous) and continue for 2–3 days postoperatively. For non-CABG surgeries (e.g., isolated valve surgery), current evidence does not support routine use. NAC has a favorable safety profile; common adverse effects include nausea and vomiting (with oral formulations), and rarely, rash and bronchospasm (with intravenous formulations). It is contraindicated in patients with aspirin-induced asthma. Based on the available evidence, although a subgroup analysis of CABG patients suggested a potential signal for POAF prevention, the overall evidence from large meta-analyses did not demonstrate consistent clinical benefit for major outcomes. Therefore, NAC is not recommended for routine perioperative use. The potential signal in the CABG subpopulation merits further investigation in prospective, adequately powered RCTs before clinical recommendation can be made.

#### 3.3.2 Vitamin C

Vitamin C is a potent water-soluble antioxidant, and its cardioprotective effects are primarily achieved through direct scavenging of reactive oxygen species (ROS), regeneration of other antioxidants (e.g., vitamin E), and inhibition of the NF-κB signaling pathway (40).

Early meta-analyses (41, 42) showed that perioperative vitamin C supplementation significantly reduced the incidence of postoperative atrial fibrillation after cardiac surgery. However, a recent high-quality randomized controlled trial did not show positive results (43). This discrepancy may be attributed to differences in multiple factors, including patient baseline nutritional status, surgical type, and the dosage and timing of vitamin C administration (42, 44). Large-scale, high-quality studies with uniform protocols are still lacking to further clarify the clinical value of vitamin C in the perioperative period of cardiac surgery.

Based on available evidence, vitamin C may be considered for patient populations at risk of vitamin C deficiency, including the elderly, malnourished individuals, and smokers. It is not recommended as a routine perioperative supplement. High-dose vitamin C may increase the risk of kidney stone formation and should be used with caution in patients with renal insufficiency. Based on the available evidence, the clinical benefit of vitamin C remains inconsistent, and the strength of recommendation is “potential benefit.”

#### 3.3.3 Melatonin

Melatonin is an endogenous neurohormone with multiple physiological functions, including potent antioxidant, anti-inflammatory, and circadian rhythm-regulating effects (45). In the perioperative period of cardiac surgery, its potential myocardial protective mechanisms mainly include: (1) directly scavenging reactive oxygen species (ROS) and inhibiting lipid peroxidation; (2) inhibiting the NF-κB signaling pathway and reducing the release of pro-inflammatory cytokines; (3) stabilizing the mitochondrial membrane structure and reducing the opening of the mitochondrial permeability transition pore (mPTP); and (4) regulating circadian rhythm and improving postoperative sleep quality.

Multiple small randomized controlled trials (46–49) have shown that perioperative melatonin supplementation improves postoperative cardiac function (ejection fraction), reduces oxidative stress markers (e.g., TNF-*α*, malondialdehyde MDA), and may reduce the incidence of postoperative atrial fibrillation. Preliminary evidence suggests that a 10 mg dose may be slightly more effective than 5 mg; however, given the small sample sizes and lack of standardized dosing regimens across studies, these conclusions should be interpreted with caution.

Based on available evidence, melatonin may be considered for the following patient populations: (1) those with preoperative sleep disorders; (2) elderly patients (endogenous melatonin secretion decreases with age); and (3) those undergoing complex cardiac surgery with anticipated severe oxidative stress and inflammatory responses. The recommended regimen is oral melatonin 5–10 mg starting on the evening before surgery and continuing for 3–5 days postoperatively. Melatonin has a favorable safety profile, with rare adverse effects (occasional dizziness, somnolence). It should be noted that melatonin may potentiate the anticoagulant effect of warfarin; thus, close monitoring of the international normalized ratio (INR) is recommended when used concomitantly. Based on the available evidence, melatonin is supported by moderate-quality evidence and has potential clinical promise, but more high-quality RCTs are needed for validation. The strength of recommendation is “consider use.”

### 3.4 Trace minerals

Trace minerals such as selenium and zinc play essential roles as cofactors for antioxidant enzymes (e.g., glutathione peroxidases) and are involved in immune function and tissue repair. Although their potential relevance to cardiac surgery outcomes is biologically plausible, dedicated perioperative RCT evidence remains extremely limited. Currently, no high-quality studies support the routine use of selenium or zinc supplementation in this setting, and they are therefore not included as individual subsections in this review. Future research is warranted to explore their roles in specific deficiency states.

### 3.5 Immunonutrition and amino acids

This category includes conditionally essential amino acids that support immune function, maintain gut barrier integrity, and modulate inflammatory responses under surgical stress. Their mechanisms extend beyond simple energy provision to include glutathione preservation and endothelial protection.

#### 3.5.1 Glutamine

Glutamine is a conditionally essential amino acid, and its body requirements significantly increase under stress conditions such as cardiac surgery (50). Its potential cardioprotective mechanisms mainly include: (1) maintaining glutathione (GSH) synthesis and enhancing endogenous antioxidant defense; (2) improving vascular endothelial function and reducing vascular permeability; (3) modulating inflammatory responses and inhibiting excessive release of inflammatory mediators; and (4) maintaining intestinal mucosal barrier integrity and reducing bacterial translocation.

Basic studies (51, 52) have shown that glutamine prevents GSH depletion and reduces the release of myocardial injury markers. A secondary analysis of the GLUTAMICS trial (53) found that intraoperative glutamate infusion significantly reduced the risk of postoperative acute kidney injury (AKI; RR = 0.49, 95%CI 0.29–0.83, *p* = 0.008). However, current guidelines from the European Society for Clinical Nutrition and Metabolism (ESPEN) do not recommend routine use of glutamine, suggesting that its clinical application still requires caution (54).

Based on available evidence, glutamine may be considered for high-risk patient populations, including those with preoperative malnutrition, those undergoing complex surgery, or those with anticipated prolonged intensive care unit (ICU) stay. The recommended regimen is intravenous supplementation initiated intraoperatively or in the early postoperative period at a dose of 0.3–0.5 g/kg/day. It should be noted that high-dose glutamine may lead to elevated blood ammonia levels and should be used with caution in patients with hepatic insufficiency. Based on the available evidence, glutamine is supported by moderate-quality evidence with potential benefit, but clinical application requires careful weighing of benefits against potential risks.

#### 3.5.2 L-Citrulline

L-Citrulline is a precursor for nitric oxide (NO) synthesis and exerts vasodilatory effects through the L-arginine-NO signaling pathway (55). In the perioperative period of cardiac surgery, its potential cardioprotective mechanisms mainly include: (1) increasing NO production and improving vascular endothelial function; (2) reducing the risk of pulmonary hypertension; and (3) inhibiting inflammatory responses and oxidative stress.

Current clinical evidence is primarily derived from the pediatric population undergoing congenital heart surgery. A study by Smith et al. (56) showed that intravenous or oral citrulline supplementation effectively maintained plasma citrulline levels, and no patient with a plasma citrulline level >37 μmol/L developed postoperative pulmonary hypertension. Another small randomized controlled trial (57) reported that no patient in the oral citrulline supplementation group developed pulmonary hypertension, whereas the incidence in the control group was 67%. In adult patients with heart failure, L-arginine or citrulline malate supplementation significantly reduced pulmonary artery pressure and improved right ventricular ejection fraction and exercise tolerance (58). However, dedicated studies in adult cardiac surgery are currently lacking.

Based on available evidence, citrulline may be considered for patients at high risk of pulmonary hypertension, including those with preexisting pulmonary hypertension, right ventricular dysfunction, or those undergoing complex congenital heart surgery. Due to the lack of direct evidence in adult cardiac surgery, extrapolation of these conclusions should be done with caution. Citrulline has a favorable safety profile, with rare adverse effects. Based on the available evidence, citrulline shows promise in preventing postoperative pulmonary hypertension after pediatric congenital heart surgery, but evidence in adult cardiac surgery is insufficient, and the strength of recommendation is “insufficient evidence.”

### 3.6 NO pathway modulators

This class of supplements enhances nitric oxide bioavailability through various mechanisms—either by providing substrate (arginine/citrulline, already covered above), by acting as an adenosine receptor agonist, or by supplying dietary nitrate that is reduced to nitrite and then to NO. These agents primarily improve endothelial function, reduce vasoconstriction, and may confer preconditioning-like protection. However, clinical translation has proven challenging.

#### 3.6.1 Adenosine

Adenosine is an endogenous purine nucleoside that mimics the effects of ischemic preconditioning by activating A1 receptors, thereby reducing myocardial infarct size and improving cardiac function (59).

A phase II randomized controlled trial by Mentzer et al. (60) showed that the addition of high-dose adenosine (2 mM) to cardioplegic solution combined with intravenous infusion significantly reduced the incidence of myocardial infarction (1.2% vs. 9.5%, *p* = 0.006) and the occurrence of composite endpoints. However, subsequent RCTs by Cohen, Laurikka, and colleagues (61) failed to replicate these benefits. An RCT by Rinne et al. (62) including 80 patients undergoing aortic valve replacement also showed negative results. Available evidence indicates that the protective effects of adenosine are highly dependent on the specific regimen (including dose, timing of administration, and combination strategies), making clinical translation challenging.

Based on available evidence, adenosine is not recommended as a routine supplement in the perioperative period of cardiac surgery. Based on the available evidence, the clinical benefit of adenosine is inconsistent, and the strength of recommendation is “not recommended for routine use.”

#### 3.6.2 Dietary nitrates/beetroot juice

Dietary nitrates (e.g., from beetroot juice) are reduced to nitrite by oral bacteria in the human body; nitrite is further converted to nitric oxide (NO) under acidic conditions, thereby exerting physiological effects including vasodilation, improved mitochondrial function, and reduced oxidative stress (63).

Animal studies have shown that dietary nitrates reduce myocardial infarct size and improve left ventricular function through mechanisms involving the H₂S and VEGFR2 signaling pathways (64). However, human randomized controlled trial evidence for the perioperative application of dietary nitrates in cardiac surgery is currently lacking.

Based on available evidence, there is currently insufficient justification for routine use of dietary nitrates/beetroot juice in the perioperative period of cardiac surgery, and their use is not recommended in clinical practice. Well-designed human RCTs are needed in the future to evaluate their efficacy and safety. Beetroot juice has a favorable safety profile but may cause harmless red discoloration of urine; high nitrate intake may interact with certain drugs (e.g., nitrates). Based on the available evidence, dietary nitrates are supported only by animal studies with no clinical studies to date, and the strength of recommendation is “not recommended for clinical use.”

### 3.7 Fat-soluble vitamins

Vitamins in this category have diverse functions beyond classic deficiency syndromes. Vitamin D exhibits immunomodulatory and vascular protective properties, while thiamine is essential for carbohydrate metabolism. However, current evidence supports supplementation only in the context of documented baseline deficiency, rather than as a universal perioperative strategy.

#### 3.7.1 Vitamin D

Vitamin D is not only involved in the regulation of calcium and phosphorus metabolism but also possesses multiple physiological functions including immunomodulation, anti-inflammatory effects, and vascular endothelial protection. Epidemiological and clinical studies have consistently shown that vitamin D deficiency is closely associated with an increased risk of cardiovascular disease.

A systematic review of 14 randomized controlled trials (65) suggested that preoperative baseline vitamin D levels are more important than postoperative supplementation. A meta-analysis of patients undergoing coronary artery bypass grafting (CABG) (66) showed that perioperative vitamin D supplementation significantly reduced the incidence of postoperative atrial fibrillation in patients with preexisting vitamin D deficiency (RR = 0.60, 95%CI 0.41–0.88, *p* = 0.01), but had no significant effect on hospital length of stay.

Based on available evidence, vitamin D is recommended for patients with baseline deficiency or insufficiency (serum 25-OH vitamin D < 30 ng/mL). In clinical practice, preoperative routine screening of vitamin D levels is recommended, and supplementation should be provided for deficient patients. The recommended regimen is oral vitamin D3 1,000–2,000 IU/day for 4–8 weeks. Routine high-dose bolus supplementation is not recommended. Vitamin D has a favorable safety profile, but long-term high-dose supplementation may lead to hypercalcemia and kidney stones. Based on the available evidence, vitamin D provides clinical benefit only for patients with baseline deficiency or insufficiency, and the strength of recommendation is “consider use” with screening-based precision supplementation.

#### 3.7.2 Thiamine (vitamin B1)

Thiamine is an essential cofactor in glucose metabolism, and its severe deficiency can lead to Wernicke’s encephalopathy and heart failure (wet beriberi). In the perioperative period of cardiac surgery, thiamine supplementation theoretically exerts myocardial protective effects by improving energy metabolism.

However, current clinical evidence does not support routine perioperative use of thiamine. A phase II randomized controlled trial by Rabie et al. (67) and an RCT by Luger et al. (68) both reported negative results, showing that thiamine supplementation did not improve clinical outcomes in cardiac surgery patients.

Based on available evidence, thiamine supplementation should only be considered in patients with confirmed thiamine deficiency, including those with chronic alcoholism, malnutrition, or malignancy. It is not recommended as a routine perioperative supplement. Thiamine has a favorable safety profile, with rare adverse effects. Based on the available evidence, thiamine RCTs have yielded negative results, and routine use is not recommended.

### 3.8 Emerging supplements (animal studies or small-scale proof-of-concept studies only)

Several nutritional compounds have shown cardioprotective effects in preclinical studies, but currently lack adequate high-quality human evidence to support their perioperative use in cardiac surgery. These include NAD + precursors (NMN/NR), pyrroloquinoline quinone (PQQ), astaxanthin, quercetin, curcumin, carnosine, and ergothioneine. The available evidence for these agents is primarily derived from animal models of ischemia–reperfusion injury or small-scale proof-of-concept studies, with no dedicated RCTs in the cardiac surgery population. A summary of these emerging supplements is provided in Table 2. Given the absence of validated clinical data, these supplements are not recommended for perioperative use in cardiac surgery outside of well-designed research protocols.

**Table 2:** Emerging supplements with preclinical evidence only—not recommended for clinical use.

Supplement	Source/Nature	Current evidence level	Clinical recommendation
NAD + precursors (NMN/NR)	Endogenous coenzyme	Animal studies only; no human cardiac surgery data (70–72)	Not recommended for clinical use
PQQ (Pyrroloquinoline Quinone)	Natural compound	Animal studies only; no human cardiac surgery data (73, 74)	Not recommended for clinical use
Astaxanthin	Carotenoid	Small human study in heart failure; no cardiac surgery RCT (75, 76)	Not recommended for clinical use
Quercetin	Flavonoid	Q-CABG trial completed, results unpublished (77)	Not recommended for clinical use
Curcumin	Polyphenol	One negative RCT in CABG (no benefit for POAF)	Not recommended for clinical use
Carnosine	Dipeptide	Animal studies only; no human cardiac surgery data (78)	Not recommended for clinical use
Ergothioneine	Amino acid derivative	Observational studies; negative ex vivo rabbit heart data (79–81)	Not recommended for clinical use

### 3.9 Supplements requiring caution or contraindication

The following supplements have clear safety concerns in the perioperative period of cardiac surgery and should be used with caution or contraindicated.

Ginseng: Ginsenosides contained in ginseng inhibit platelet aggregation, thereby increasing the risk of perioperative bleeding. Therefore, discontinuation of ginseng preparations before surgery is recommended, and the specific timing of discontinuation should be determined based on the drug half-life and the recovery period of platelet function.

Red Yeast Rice: Red yeast rice contains natural statin components (lovastatin analogs), and concomitant use with statin drugs may increase the risk of myopathy and rhabdomyolysis (69). Discontinuation of red yeast rice preparations 5–7 days before surgery is recommended to reduce the risk of perioperative drug interactions.

In summary, due to the clear risks of bleeding, drug interactions, or infection associated with the above supplements, the necessity of their use should be carefully evaluated during the perioperative period of cardiac surgery, and they should be discontinued in advance when necessary. Based on the available evidence, routine use is not recommended.

## 4 Evidence-based reference framework for clinical decision-making

In the absence of a formal systematic review, the recommendation grading in Table 1 is based on a narrative synthesis of available evidence, integrating considerations of evidence quality (derived from published meta-analyses and RCTs), clinical benefit, and safety. This framework is intended to provide clinicians with a practical, evidence-informed reference rather than a formal guideline.

**Figure 2:**
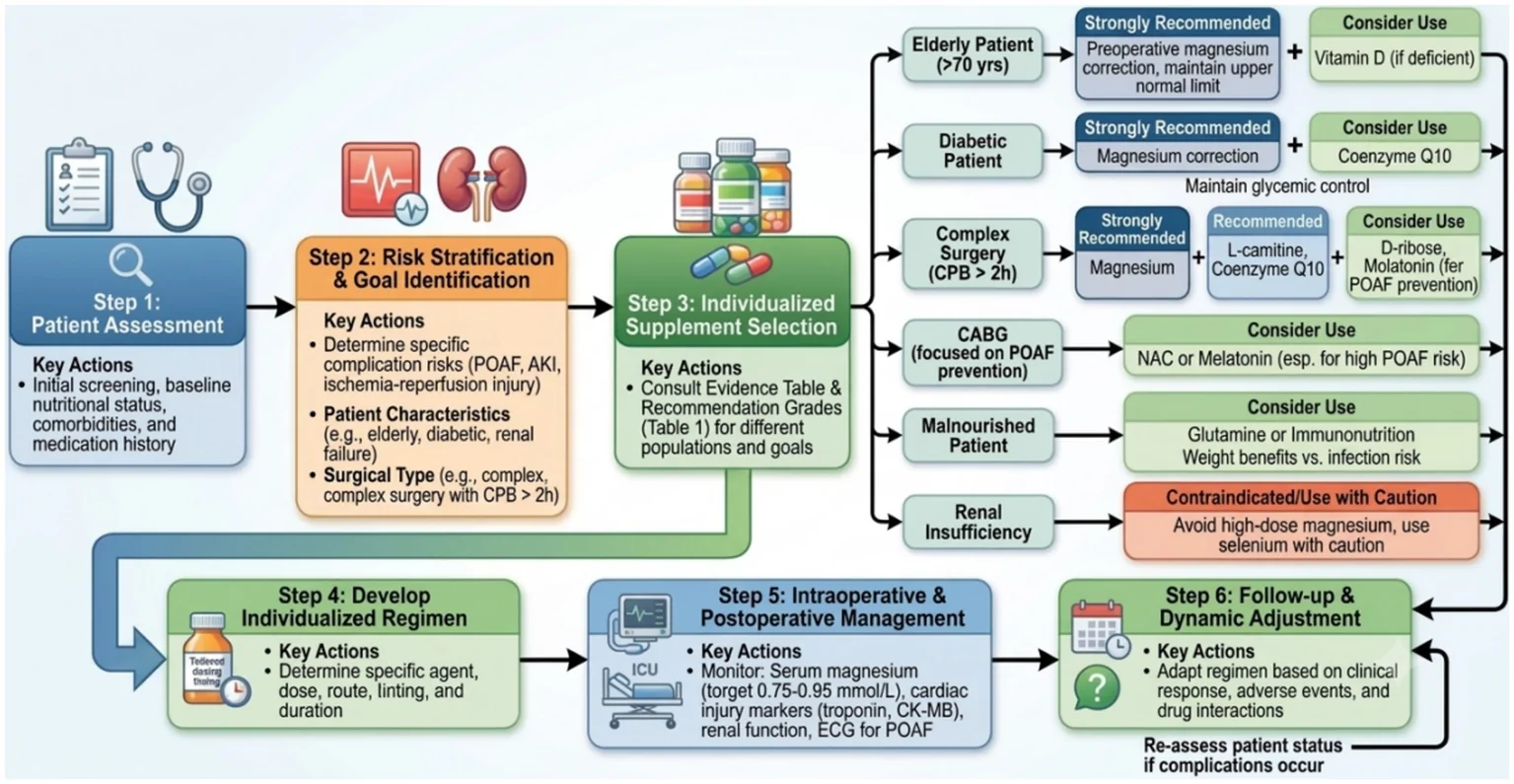
Flow diagram of the clinical decision-making framework.

Based on available evidence, this section provides an evidence-based reference framework for the use of nutritional supplements in the perioperative period of cardiac surgery (Figure 2). Rather than offering definitive precision algorithms, this framework is intended to help clinicians quickly identify supplements with different levels of evidence and consider patient-specific factors when making individualized decisions. The framework considers the following four dimensions: (1) the evidence level of supplements (Table 1); (2) patient-specific characteristics, including age, sex, baseline nutritional status, and comorbidities; (3) surgical type and risk stratification, covering CABG, valve surgery, great vessel surgery, as well as CPB duration and surgical complexity; and (4) anticipated benefits and potential risks, including efficacy, adverse effects, drug interactions, and cost-effectiveness.

The decision-making process consists of five steps: Step 1: Assess patient baseline status, including nutritional screening, biochemical indicator testing, and medication history; Step 2: Based on surgical type and risk stratification, determine the complications that require focused attention (e.g., CABG patients should focus on POAF and AKI); Step 3: Based on evidence level and patient characteristics, select supplements with higher recommendation grades (e.g., magnesium, L-carnitine, coenzyme Q10); Step 4: Develop an individualized regimen specifying the type, dose, route of administration, timing, and duration of supplementation; Step 5: Conduct intraoperative monitoring and postoperative follow-up. Step 6: Dynamically adjust the regimen based on clinical response.

### 4.1 Recommendation grading criteria

Referring to the GRADE system, and considering the quality of evidence-based data and the degree of clinical benefit, perioperative nutritional supplements are classified into the following recommendation grades (Table 1).

### 4.2 Patient-specific considerations for supplement selection

Based on the characteristics of different patient populations, the following patient-specific considerations may inform clinical decision-making: For elderly patients (>70 years), priority should be given to magnesium and vitamin D after screening, with attention to potential drug interactions; diabetic patients often have hypomagnesemia and endothelial dysfunction, so magnesium combined with coenzyme Q10 is recommended, along with enhanced blood glucose monitoring; patients with renal insufficiency should avoid high-dose magnesium and use selenium supplements with caution; malnourished patients may consider glutamine or immunonutrition support, but the risk of infection should be weighed; for patients undergoing complex surgery with an anticipated CPB duration exceeding 2 h, a combination regimen such as magnesium combined with L-carnitine and coenzyme Q10 is recommended to achieve synergistic myocardial protective effects.

## 5 Safety, interactions, and contraindications

### 5.1 Adverse events

Although nutritional supplements are generally safe, the following potential adverse events warrant attention. Magnesium may cause hypermagnesemia (especially in patients with renal insufficiency) and diarrhea (with oral formulations); L-carnitine occasionally causes nausea, abdominal pain, and body odor change (fishy odor); coenzyme Q10 may cause nausea, diarrhea, and decreased appetite, and may potentiate the anticoagulant effect of warfarin; NAC oral formulations commonly cause nausea and vomiting, while intravenous formulations rarely cause bronchospasm; high-dose or long-term vitamin C may increase the risk of kidney stones and should be used with caution in patients with renal insufficiency; melatonin may cause dizziness and somnolence, and may potentiate the anticoagulant effect of warfarin.

### 5.2 Drug interactions

Nutritional supplements may interact with commonly used cardiovascular drugs. Coenzyme Q10, melatonin, and vitamin E may potentiate the anticoagulant effect of warfarin, and INR monitoring is recommended when used concomitantly. Ginseng increases bleeding risk by inhibiting platelet aggregation, and discontinuation at least 7 days before surgery is recommended. Red yeast rice contains natural statin components (lovastatin analogs), and concomitant use with statins may increase the risk of myopathy; discontinuation 5–7 days before surgery is recommended. High-dose glutamine may lead to elevated blood ammonia levels and should be used with caution in patients with hepatic insufficiency.

### 5.3 Monitoring indicators

When using nutritional supplements during the perioperative period, the following indicators are recommended for monitoring: (1) serum magnesium levels (for those using magnesium); (2) myocardial injury markers, such as cardiac troponin and CK-MB; (3) renal function indicators, including creatinine, blood urea nitrogen, and urine output; (4) electrocardiogram for monitoring arrhythmias, especially POAF; (5) complete blood count and liver function (with long-term or high-dose use); and (6) INR (for patients on warfarin concomitantly receiving coenzyme Q10, melatonin, or vitamin E).

## 6 Conclusion and future directions

This review systematically evaluates the evidence base for commonly used oral nutritional supplements in the perioperative period of cardiac surgery. The main findings are as follows: Magnesium has the most robust evidence and the highest recommendation level and should be considered standard adjunctive therapy in the perioperative period of cardiac surgery; L-carnitine and coenzyme Q10 are supported by moderate-quality evidence with clear clinical benefit and are recommended for specific patient populations; Vitamin C, NAC, melatonin, D-ribose, citrulline, and glutamine have shown promising efficacy but require further validation through high-quality RCTs; Recent RCTs of creatine/phosphocreatine, taurine, and thiamine have shown negative results or insufficient evidence, and routine use is not recommended; Emerging supplements (NAD + precursors, PQQ, astaxanthin, quercetin, curcumin, carnosine, ergothioneine) currently have only animal experimental evidence, and clinical translation remains a future endeavor; Individualized intervention is critical, as efficacy varies significantly across different patient populations, surgical types, and baseline statuses, necessitating individualized selection.

All cardiac surgery patients should routinely receive magnesium supplementation (unless contraindicated), with preoperative correction of hypomagnesemia; For complex cardiac surgery (anticipated CPB duration >2 h) or patients with poor preoperative cardiac function, combination therapy with L-carnitine and coenzyme Q10 is recommended; For CABG patients, especially those at high risk for POAF (elderly, history of atrial fibrillation, left atrial enlargement), NAC or melatonin may be considered for prevention; Routine use of supplements with insufficient evidence such as creatine/phosphocreatine, taurine, and thiamine is not recommended; Ginseng and red yeast rice, which may affect coagulation or interact with statins, should be used with caution; We recommend that clinicians consider patient characteristics, surgical type, and baseline status when selecting supplements, using the evidence-based reference framework provided in this review as a guide. The development of truly precision-based approaches—grounded in biomarkers, genetic profiles, and validated predictive tools—remains an important direction for future research.

Future research should focus on the following directions: Conducting more high-quality, large-sample, multicenter RCTs to validate the efficacy of promising supplements (e.g., L-carnitine, CoQ10, melatonin); Exploring formulation technologies to improve bioavailability (e.g., nano-formulations, liposomal encapsulation, sustained-release formulations) to enhance supplement absorption and duration of action; Establishing precision nutrition intervention strategies based on patient genotype (e.g., drug-metabolizing enzyme polymorphisms), phenotype (e.g., baseline nutritional status, biochemical markers), and surgical characteristics; Promoting translational research to advance promising supplements from animal studies (e.g., NAD + precursors, PQQ, astaxanthin) to human clinical trials; Investigating the synergistic effects and safety of combination regimens with multiple supplements to establish rational combination protocols; Establishing a safety monitoring database for perioperative nutritional supplement use to accumulate real-world evidence.

## References

[1] Banerjee D Feng Y Morse B. Strategies to attenuate maladaptive inflammatory response associated with cardiopulmonary bypass. Front Surg. (2024) 11:1224068. 39022594 10.3389/fsurg.2024.1224068 PMC11251955

[2] Gerster PA Klesse A Chang J Erb JM Goettel N. Neurological complications in cardiac surgery. Curr Anesthesiol Rep. (2019) 9:223–33. doi: 10.1007/s40140-019-00344-x

[3] Saetang M Wasinwong W Oofuvong M. Effect of combined vitamin C and thiamine therapy on myocardial and inflammatory markers in cardiac surgery: a randomized controlled clinical trial. Nutrients. (2025) 17:1006. doi: 10.3390/nu17061006, 40290059 PMC11944524

[4] Abbasciano RG Tomassini S Roman MA Rizzello A Pathak S Ramzi J . Effects of interventions targeting the systemic inflammatory response to cardiac surgery on clinical outcomes in adults. Cochrane Database Syst Rev. (2023) 10:CD013584. doi: 10.1002/14651858.CD013584.pub2, 37873947 PMC10594589

[5] Raja SG Benedetto U Marczin N. Editorial: inflammation and heart surgery. Front Cardiovasc Med. (2024) 11:1493898. doi: 10.3389/fcvm.2024.1493898, 39359638 PMC11445150

[6] Alsadder L Hamadah A. Cardiac ischaemia–reperfusion injury: pathophysiology, therapeutic targets and future interventions. Biomedicine. (2025) 13:2084. doi: 10.3390/biomedicines13092084, 41007647 PMC12467651

[7] Sabe SA Harris DD Broadwin M. Cardioprotection in cardiovascular surgery. Basic Res Cardiol. (2024) 119:545–68. doi: 10.1007/s00395-024-01062-0, 38856733 PMC12978342

[8] Yuan S Che Y Wang Z. Mitochondrion-targeted carboxymethyl chitosan hybrid nanoparticles loaded with coenzyme Q10 protect cardiac grafts against cold ischaemia–reperfusion injury in heart transplantation. J Transl Med. (2023) 21:925. doi: 10.1186/s12967-023-04763-7, 38124174 PMC10734076

[9] Robitaille M Ahmed U Valencia J Khan A Manji AA Russ E . Impaired PGC-1α-pAMPK signaling in postmenopausal women undergoing cardiac surgery and the role of nicotinamide in its reversal: insights from a murine model. J Mol Cell Cardiol Plus. (2025) 14:100831. 41377471 10.1016/j.jmccpl.2025.100831 PMC12686929

[10] Rabanal-Ruiz Y Llanos-González E Alcain FJ. The use of coenzyme Q10 in cardiovascular diseases. Antioxidants. (2021) 10:755. doi: 10.3390/antiox10050755, 34068578 PMC8151454

[11] Curko-Cofek B Jenko M Taleska Stupica G. The crucial triad: endothelial glycocalyx, oxidative stress, and inflammation in cardiac surgery—exploring the molecular connections. Int J Mol Sci. (2024) 25:10891. doi: 10.3390/ijms252010891, 39456673 PMC11508174

[12] Zhang J Zhang H Wang J Wang T Bian L Li Z . The organ-protective effects of nitric oxide in adult patients undergoing cardiac surgery with cardiopulmonary bypass: a systematic review and meta-analysis. BMC Anesthesiol. (2025) 25:335. 40610908 10.1186/s12871-025-03207-7 PMC12232135

[13] Papagiannidou A Mitropoulou M Papantzikos K. Hypomagnesemia: a clinical and nutritional update. Curr Nutr Rep. (2026) 15:30. doi: 10.1007/s13668-026-00745-5, 41872423 PMC13009017

[14] Liu T Wang L Pei S. Biological functions and detection strategies of magnesium ions: from basic necessity to precise analysis. Analyst. (2025) 150:2979–99. doi: 10.1039/D5AN00425J, 40539939

[15] Stanojević M Djuricic N Parezanovic M. The impact of chronic magnesium deficiency on excitable tissues—translational aspects. Biol Trace Elem Res. (2025) 203:707–28. doi: 10.1007/s12011-024-04216-2, 38709369

[16] Salaminia S Sayehmiri F Angha P. Evaluating the effect of magnesium supplementation and cardiac arrhythmias after acute coronary syndrome: a systematic review and meta-analysis. BMC Cardiovasc Disord. (2018) 18:129. doi: 10.1186/s12872-018-0857-6, 29954320 PMC6025730

[17] Shiga T Wajima Z Inoue T Ogawa R. Magnesium prophylaxis for arrhythmias after cardiac surgery: a meta-analysis. Am J Med. (2004) 117:325–33. 15336582 10.1016/j.amjmed.2004.03.030

[18] Miller S Crystal E Garfinkle M Lau C Lashevsky I Connolly SJ. Oral magnesium supplementation reduces atrial fibrillation after cardiac surgery: a meta-analysis. Ann Thorac Surg. (2005) 79:1538–42. 10.1136/hrt.2004.033811 PMC1768903 15831645

[19] Shalabi L Ibrahim A Adel Elsawy M Elsawy MA Zreigh S Dervis M . L-carnitine supplementation to prevent postoperative complications after cardiac surgery: a systematic review and meta-analysis of randomised clinical trials. Indian J Anaesth. (2025) 69:547–60. doi: 10.4103/ija.ija_1325_24, 40470390 PMC12133040

[20] Werida RH Okda SM. Therapeutic potential of L-carnitine in coronary artery disease: a systematic review. Inflammopharmacology. (2026) 34:1331–42. doi: 10.1007/s10787-025-02048-7, 41709059 PMC12995981

[21] Furat C İlhan G Bayar E Bozok Ş Güvener M Yılmaz M. L-carnitine on myocardial function after coronary artery bypass grafting. Turk Gogus Kalp Damar Cerrahisi Derg. (2018) 26:22–9. doi: 10.5606/tgkdc.dergisi.2018.14620, 32082707 PMC7018104

[22] Crane FL. Biochemical functions of coenzyme Q10. J Am Coll Nutr. (2001) 20:591–8. doi: 10.1080/07315724.2001.10719063, 11771674

[23] Rosenfeldt FL Marasco S Lyon W Wowk M Sheeran F Bailey M . Coenzyme Q10 therapy before cardiac surgery improves mitochondrial function and in vitro contractility of myocardial tissue. J Thorac Cardiovasc Surg. (2005) 129:25–32. doi: 10.1016/j.jtcvs.2004.03.034, 15632821

[24] Makhija N Sendasgupta C Kiran U Lakshmy R Hote MP Choudhary SK . The role of oral coenzyme Q10 in patients undergoing coronary artery bypass graft surgery. J Cardiothorac Vasc Anesth. (2008) 22:832–9. doi: 10.1053/j.jvca.2008.03.007, 18834786

[25] Chello M Mastroroberto P Romano R Bevacqua E Pantaleo D Ascione R . Protection by coenzyme Q10 from myocardial reperfusion injury during coronary artery bypass grafting. Ann Thorac Surg. (1994) 58:1427–32. 7979670 10.1016/0003-4975(94)91928-3

[26] Taggart DP Jenkins M Hooper J Hadjinikolas L Kemp M Hue D . Effects of intravenous coenzyme Q10 on left ventricular function in patients with coronary artery disease. Ann Thorac Surg. (1996) 61:829–33. 8619701 10.1016/0003-4975(95)01120-X

[27] Omran H Illien S MacCarter D Cyr J St. Lüderitz B. D-ribose improves diastolic function and quality of life in congestive heart failure patients. Eur J Heart Fail. (2003) 5:615–9. 14607200 10.1016/s1388-9842(03)00060-6

[28] Wyatt DA Ely SW Walsh R Mainwaring R Berne RM Lasley RD . Ventricular functional restoration with ATP precursors after hypothermic cardioplegic ischemia. J Thorac Cardiovasc Surg. (1988) 96:238–45.

[29] MacCarter D Vijay N Washam M Shecterle L Sierminski H Cyr JA St.. D-ribose administration in patients undergoing aortic valve replacement. FASEB J. (2000) 14:A398.

[30] Perkowski DJ Wagner S Schneider JR Cyr JA St.. A targeted metabolic protocol with D-ribose for off-pump coronary artery bypass procedures: a retrospective analysis. J Extra Corpor Technol. (2011) 43:150–6. 10.1177/1753944711412421 21693564

[31] Schlattner U Klaus A Ramirez Rios S Guzun R Kay L Tokarska-Schlattner M. Cellular compartmentation of energy metabolism and creatine kinase in health and disease. Prog Nucleic Acid Res Mol Biol. (2006) 81:173–204.

[32] Lomivorotov V Merekin D Fominskiy E. Myocardial protection with phosphocreatine in high-risk cardiac surgery patients: a randomized trial. BMC Anesthesiol. (2023) 23:389. doi: 10.1186/s12871-023-02341-4, 38030971 PMC10685505

[33] Venturini A Ascione R Lin H Polesel E Angelini GD Suleiman MS. The importance of myocardial amino acids during ischemia and reperfusion in dilated left ventricle. Exp Biol Med. (2010) 235:696–705. 10.1007/s11010-009-0101-x PMC2850556 19363596

[34] Jin G Kataoka Y Tanaka M Mizuma H Nozaki S Tahara T . Changes in tissue amino acid concentrations in an in vivo model of open heart surgery. J Mol Cell Cardiol. (1998) 30:803–12.9602429

[35] Schaffer SW Jong CJ KC R Azuma J. Amino acids in the heart and the role of taurine in the cardiomyocyte. Exp Biol Med. (2008) 233:693–701.

[36] Takatani T Takahashi K Uozumi Y Matsuda T Ito T Schaffer SW . Taurine prevents the ischemia-induced apoptosis in cultured neonatal rat cardiomyocytes through Akt/caspase-9 pathway. Biol Pharm Bull. (2004) 27:497–503. 10.1016/j.bbrc.2004.02.066 15020243

[37] Rushworth GF Megson IL. Existing and potential therapeutic uses for N-acetylcysteine: the need for conversion to intracellular glutathione for antioxidant benefits. Pharmacol Ther. (2014) 141:150–9. doi: 10.1016/j.pharmthera.2013.09.006, 24080471

[38] Pereira JEG El Dib R Braz LG Escudero J Hayes J . N-acetylcysteine use among patients undergoing cardiac surgery: a systematic review and meta-analysis of randomized trials. PLoS One. (2019) 14:e0213862. 31071081 10.1371/journal.pone.0213862 PMC6508704

[39] Hanafy DA Willim HA Suwatri WT. Efficacy of N-acetylcysteine for prevention of postoperative atrial fibrillation following coronary artery bypass grafting: a systematic review and Meta-analysis of randomized controlled trials. Rev Cardiovasc Med. (2024) 25:243. doi: 10.31083/j.rcm2507243, 39139444 PMC11317354

[40] May JM. Vitamin C transport and its role in the central nervous system. Subcell Biochem. (2007) 43:193–217. 10.1007/978-94-007-2199-9_6 PMC3725125 22116696

[41] Shi R Li ZH Chen D Wu QC Zhou XL Tie HT. Sole and combined vitamin C supplementation can prevent postoperative atrial fibrillation after cardiac surgery: a systematic review and meta-analysis. Clin Cardiol. (2019) 42:674–9. 10.1002/clc.22951 PMC6489881 29603289

[42] Hu X Yuan L Wang H Li C Cai J Hu Y . Efficacy and safety of vitamin C for atrial fibrillation after cardiac surgery: a meta-analysis with trial sequential analysis. Int J Surg. (2017) 37:58–64. 27956113 10.1016/j.ijsu.2016.12.009

[43] Caldonazo T Kirov H Rahouma M Robinson NB Demetres M Gaudino M . Vitamin C and atrial fibrillation after cardiac surgery: a systematic review and meta-analysis. J Thorac Cardiovasc Surg. (2017) 153:794–802. 10.1016/j.jtcvs.2021.03.077 33952399

[44] Hu X Yuan L Wang H Li C Cai J Hu Y . Effect of vitamin C on postoperative atrial fibrillation after cardiac surgery: a meta-analysis. Am Heart J. (2013) 166:527–32.24016503

[45] Tan DX Chen LD Poeggeler B Manchester LC Reiter RJ. Melatonin: a potent, endogenous hydroxyl radical scavenger. Endocr J. (1993) 1:57–60.

[46] Mohammadi N Alizadeh M Akbarzadeh S. Melatonin administered postoperatively lowers oxidative stress and inflammation and significantly recovers heart function in patients undergoing CABG surgery. Eur J Med Res. (2025) 30:585. doi: 10.1186/s40001-025-02789-9, 40619439 PMC12232798

[47] Jouybar R Khademi S Razmjooie S Bagheri N. Effect of preoperative melatonin on myocardial ischemia-reperfusion injury following elective CABG surgery. J Cardiothorac Vasc Anesth. (2022) 36:2829–36.35144871

[48] Dwaich KH FGY Al-Amran BIM AL-Sheibani Al-Aubaidy HA. Melatonin improves heart function and prevents oxidative stress and apoptosis in cardiomyocytes following ischemia-reperfusion injury in rats. J Cardiovasc Pharmacol. (2022) 80:547–55.35522143

[49] Zarezadeh M Khorshidi M Emami M Janmohammadi P Kord-varkaneh H Mousavi SM . Melatonin supplementation and pro-inflammatory mediators after cardiac surgery: a systematic review and meta-analysis of randomized controlled trials. Front Cardiovasc Med. (2022) 9:832591.35295271

[50] Hausenloy DJ Ong SB Yellon DM. The mitochondrial permeability transition pore as a target for preconditioning and postconditioning. Basic Res Cardiol. (2007) 102:265–73. 19242644 10.1007/s00395-009-0010-x

[51] Engel JM Mühling J Kwapisz M Heidt M. Glutamine administration in patients undergoing cardiac surgery and the influence on blood glutathione levels. Acta Anaesthesiol Scand. (2009) 53:1317–23. doi: 10.1111/j.1399-6576.2009.02084.x, 19681775

[52] Sufit A Weitzel LB Hamiel C Queensland K Dauber I Rooyackers O . Pharmacologically dosed oral glutamine reduces myocardial injury in patients undergoing cardiac surgery. JPEN. (2012) 36:556–61. doi: 10.1177/0148607112448823, 22623413

[53] Holm J Vanky F Svedjeholm R. Association of Glutamate Infusion with Risk of acute kidney injury after coronary artery bypass surgery: a pooled analysis of 2 randomized clinical trials. JAMA Netw Open. (2024) 7:e2351743. doi: 10.1001/jamanetworkopen.2023.51743, 38252440 PMC10804267

[54] Weimann A Braga M Carli F Higashiguchi T Hübner M Klek S . ESPEN practical guideline: clinical nutrition in surgery. Clin Nutr. (2021) 40:4745–61. doi: 10.1016/j.clnu.2021.03.031, 34242915

[55] Ruiz S Grosso C Tablada M. Efficacy of citrulline supplementation to decrease the risk of pulmonary hypertension after congenital heart disease surgery: a local experience. Rev Fac Cien Med Univ Nac Cordoba. (2020) 77:249–53. 33351387 10.31053/1853.0605.v77.n4.27936

[56] Orozco-Gutierrez JJ Castillo-Martinez L Orea-Tejeda A Vazquez-Diaz O Valdespino-Trejo A Narvaez-David R . Effect of L-arginine or L-citrulline oral supplementation on blood pressure and right ventricular function in heart failure patients with preserved ejection fraction. Cardiol J. (2010) 17:612–8. 21154265

[57] Lasley RD Shryock JC Belardinelli L. Adenosine and adenosine receptors in ischemic preconditioning. J Pharmacol Exp Ther. (1999) 289:637–44.

[58] Mentzer RM Jr Birjiniuk V Khuri S Lowe JE Rahko PS Weisel RD . Adenosine myocardial protection: preliminary results of a phase II clinical trial. Ann Surg. (1999) 229:643–9. 10235522 10.1097/00000658-199905000-00006 PMC1420808

[59] Layland J Carrick D Lee M Oldroyd K Berry C. Adenosine in cardioplegia: a systematic review of the literature. Eur J Cardiothorac Surg. (2003) 23:469–76.

[60] Rinne T Laurikka J Penttilä I Kaukinen S. Adenosine in cold blood cardioplegia – a placebo-controlled study. Eur J Cardiothorac Surg. (2000) 18:582–8. 10.1016/s1053-0770(00)90049-1 10698386

[61] Larsen FJ Weitzberg E Lundberg JO Ekblom B. Dietary nitrate reduces maximal oxygen consumption while maintaining work performance in maximal exercise. Free Radic Biol Med. (2010) 48:342–7. doi: 10.1016/j.freeradbiomed.2009.11.006, 19913611

[62] Salloum FN Sturz GR Yin C. Beetroot juice reduces infarct size and improves cardiac function following ischemia-reperfusion injury: possible involvement of endogenous H2S. Exp Biol Med (Maywood). (2015) 240:669–81. doi: 10.1177/1535370214558024, 25361774 PMC4935262

[63] Dohain AM Almogati J Al-Radi OO Elassal AA Zaher ZF Fatani TH . Vitamin D deficiency and postoperative outcomes in cardiac surgery. Ann Thorac Surg. (2015) 100:2187–93.

[64] Ansari SA Dhaliwal J Ansari Y. The role of vitamin D supplementation before coronary artery bypass grafting in preventing postoperative atrial fibrillation in patients with vitamin D deficiency or insufficiency: a systematic review and meta-analysis. Cureus. (2023) 15:e36496. doi: 10.7759/cureus.36496, 37090368 PMC10119034

[65] Andersen LW Holmberg MJ Berg KM. Thiamine as an adjunctive therapy in cardiac surgery: a randomized, double-blind, placebo-controlled, phase II trial. Crit Care. (2016) 20:92. doi: 10.1186/s13054-016-1245-1, 27044557 PMC4820988

[66] Luger M Hiesmayr M Köppel P Sima B Ranz I Weiss C . Perioperative thiamine administration in cardiac surgery: a randomized controlled trial. Ann Thorac Surg. (2017) 104:1574–81.

[67] Donnino MW Carney E Cocchi MN Barbash I Chase M Joyce N . Thiamine deficiency in critically ill patients with sepsis. J Crit Care. (2010) 25:576–81. doi: 10.1016/j.jcrc.2010.03.003, 20646908

[68] Imai S Guarente L. NAD+ and sirtuins in aging and disease. Trends Cell Biol. (2014) 24:464–71. doi: 10.1016/j.tcb.2014.04.002, 24786309 PMC4112140

[69] Wang X Li S Huo D Li C Zhang Q. Case series of bacteremia associated with probiotic use in children after cardiac surgery, China. Emerg Infect Dis. (2026) 32:28–33. doi: 10.3201/eid3201.250298, 41612523 PMC12870080

[70] Zhong O Wang J Tan Y Lei X Tang Z. Effects of NAD+ precursor supplementation on cardiac hemodynamics during ischemia-reperfusion. J Cardiovasc Pharmacol. (2021) 77:536–44.33760801

[71] Harris CB Chowanadisai W Mishchuk DO Satre MA Slupsky CM Rucker RB. Dietary pyrroloquinoline quinone (PQQ) and its role in human health. J Nutr Biochem. (2013) 24:2076–84. 24231099 10.1016/j.jnutbio.2013.07.008

[72] Zhu BQ Zhou HZ Teerlink JR. Pyrroloquinoline quinone (PQQ) decreases myocardial infarct size and improves cardiac function in rat models of ischemia and ischemia/reperfusion. Cardiovasc Drugs Ther. (2004) 18:421–31. doi: 10.1007/s10557-004-6219-x, 15770429

[73] Xu X Chen C Lu WJ Su Y-L Shi J-Y Liu Y-C . Pyrroloquinoline quinone can prevent chronic heart failure by regulating mitochondrial function. Cardiovasc Diagn Ther. (2020) 10:453–69. doi: 10.21037/cdt-20-129, 32695625 PMC7369269

[74] Mohammadi SG Shafie D Feizi A. Impact of astaxanthin on oxidative markers, uric acid, and clinical symptoms in heart failure: a randomized clinical trial. BMC Cardiovasc Disord. (2025) 25:779. doi: 10.1186/s12872-025-05260-z, 41162864 PMC12574148

[75] Dagher O Mury P Noly PE Fortier A Lettre G Thorin E . Design of a randomized placebo-controlled trial to evaluate the anti-inflammatory and senolytic effects of quercetin in patients undergoing coronary artery bypass graft surgery. Front Cardiovasc Med. (2021) 8:741542. doi: 10.3389/fcvm.2021.741542, 34746258 PMC8564044

[76] Zhao J Conklin DJ Guo Y Zhang X Obal D Guo L . Cardiospecific overexpression of ATPGD1 (carnosine synthase) increases histidine dipeptide levels and prevents myocardial ischemia reperfusion injury. J Am Heart Assoc. (2020) 9:e015222. doi: 10.1161/JAHA.119.015222, 32515247 PMC7429021

[77] Einar S Filip O Sophie H. Ergothioneine is associated with reduced mortality and decreased risk of cardiovascular disease. BMJ. (2020) 23:236-236. 10.1136/heartjnl-2019-315485 PMC7229907 31672783

[78] Cargnoni A Bernocchi P Ceconi C Curello S Ferrari R. “In vitro ergothioneine administration failed to protect isolated ischaemic and reperfused rabbit heart” in *Free Radicals in Diagnostic Medicine. Advances in Experimental Medicine and Biology*. Ed. D. Armstrong (Boston: Springer). (1994) 366. doi: 10.1007/978-1-4615-1833-4_49 7771289

[79] Tian X Thorne J Moore JB. Ergothioneine: an underrecognised dietary micronutrient required for healthy ageing? Br J Nutr. (2023) 129:104–14. doi: 10.1017/S0007114522003592, 38018890 PMC9816654

[80] Viviano A Steele D Edsell M Jahangiri M. Over-the-counter natural products in cardiac surgery: a case of ginseng-related massive perioperative bleeding. BMJ Case Rep. (2017) 64:1057–63. 10.1136/bcr-2016-218068 PMC5612204 28784871

[81] Chen T-L Yeh C-C Lin C-S Shih C-C Liao C-C. Effects of red yeast rice prescription (LipoCol forte) on adverse outcomes of surgery. QJM: Int J Med. (2019) 112:253–9. doi: 10.1093/qjmed/hcy278, 30496589

